# Clinical and radiological characteristics of patients with late-onset severe restrictive lung defect after hematopoietic stem cell transplantation

**DOI:** 10.1186/s12890-017-0466-7

**Published:** 2017-09-07

**Authors:** Ho Namkoong, Makoto Ishii, Takehiko Mori, Hiroaki Sugiura, Sadatomo Tasaka, Masatoshi Sakurai, Yuya Koda, Jun Kato, Naoki Hasegawa, Shinichiro Okamoto, Tomoko Betsuyaku

**Affiliations:** 1 Division of Pulmonary Medicine, Department of Medicine, Keio University School of Medicine, 35 Shinanomachi, Shinjuku-ku, Tokyo, 160-8582 Japan; 2 Division of Hematology, Department of Medicine, Keio University School of Medicine, Tokyo, Japan; 3 Center for Infectious Diseases and Infection Control, Keio University School of Medicine, Tokyo, Japan; 4 Department of Diagnostic Radiology, Keio University School of Medicine, Tokyo, Japan; 5 Department of Respiratory Medicine, Hirosaki University Graduate School of Medicine, Hirosaki, Japan

**Keywords:** Hematopoietic stem cell transplantation, Late-onset noninfectious pulmonary complications, Pleuroparenchymal fibroelastosis, Idiopathic pneumonia syndrome

## Abstract

**Background:**

Late-onset noninfectious pulmonary complications (LONIPCs), which occur more than 3 months after allogeneic hematopoietic stem cell transplantation (HSCT), are major causes of morbidity and mortality after transplantation. Among LONIPCs, we occasionally treat patients with late-onset severe restrictive lung defect after HSCT; however, its clinical features have not been fully elucidated.

**Methods:**

A retrospective chart review of a single center on cases of late-onset severe restrictive lung defect after HSCT was performed. Among 453 patients who survived longer than 100 days after allogeneic HSCT with evaluable spirometry data, 12 patients (2.6%) developed late-onset severe restrictive lung defect (i.e., vital capacity percent of predicted less than 60%).

**Results:**

Median duration from transplantation to diagnosis of late-onset severe restrictive lung defect cases was 44.5 months. Major computed tomography (CT) finding was pleuroparenchymal thickening with volume loss, an evidence of fibrosis, predominantly in upper lobes (*n* = 7), which was consistent with pleuroparenchymal fibroelastosis. The remaining patients showed unclassifiable interstitial pneumonia pattern (*n* = 2) and airway-predominant pattern (*n* = 3). The diffusing capacity for carbon oxide tended to decrease, while the residual volume/total lung capacity ratio tended to increase after HSCT. Of 12 patients, 8 patients died and the median month from diagnosis to death was 33.5 months. Seven patients died of pulmonary or systemic infection, and one patient died due to relapse of the primary disease.

**Conclusion:**

Severe restrictive lung defect could develop in selected cases in the late-phase after HSCT and could be a unique clinical entity with specific radiographical findings.

**Electronic supplementary material:**

The online version of this article (10.1186/s12890-017-0466-7) contains supplementary material, which is available to authorized users.

## Background

Despite recent progress in allogeneic hematopoietic stem cell transplantation (HSCT) for hematological diseases, pulmonary complications have been recognized as one of the major causes of morbidity and mortality after allogeneic HSCT, occurring in about 40–70% of patients, with a high mortality rate [1–3]. Pulmonary complications after HSCT are caused by various noninfectious and infectious causes, and develop in both the early and late phases after transplantation, contingent on the day of development before or after 100 days following HSCT.

Opportunistic infection caused by bacteria, fungus, and viruses represents a major cause of infectious pulmonary complications in the early phase after HSCT [2, 4]. In contrast, noninfectious pulmonary complications are major causes of morbidity and mortality later than 100 days after allogeneic HSCT, which have been labelled late-onset noninfectious pulmonary complications (LONIPCs) [5]. LONIPCs classically include bronchiolitis obliterans (BO), cryptogenic organizing pneumonia (COP) (previously called bronchiolitis obliterans organizing pneumonia [BOOP]), diffuse alveolar hemorrhage (DAH), and idiopathic pneumonia syndrome (IPS) [6, 7].

IPS is a severe pulmonary complication occurring after HSCT, which was originally characterized by symptoms and signs of pneumonia, restrictive pulmonary function test abnormality, and alveolar injury without documented lower respiratory tract infection [8]. The American Thoracic Society (ATS) Research Statement on IPS provides a comprehensive review of IPS, the definition of which has been updated [9]. IPS is clinically defined by three criteria: widespread alveolar injury, absence of documented infection, and absence of cardiac, renal, or iatrogenic etiology. The evidence of widespread alveolar injury is based on multilobar infiltrates on chest radiographs or computed tomography (CT), symptoms and signs of pneumonia, and evidence of abnormal pulmonary physiology, including restrictive pulmonary function test abnormality. The time of onset for IPS ranges from 4 to 106 days and diffuse alveolar hemorrhage (DAH) occurs in early post-HSCT, with an indicated median onset time of 12–15 days [9]. Therefore, it seems that LONIPCs generally do not include IPS, according to the ATS statement.

Pleuroparenchymal fibroelastosis (PPFE), originally reported as an idiopathic disease [10], has previously been reported as a novel radiological and pathological feature in patients with pulmonary disease in the late phase after HSCT [11]. However, detailed clinical and radiological features of pulmonary complications after HSCT, especially in the late phase, are still largely unknown.

The primary aim of this retrospective study was to clarify the clinical and radiological features of late-onset severe restrictive lung defect after HSCT.

## Methods

### Patients

The retrospective study protocol was approved by the Institutional Review Board of Keio University School of Medicine (Tokyo, Japan).

**Fig. 1:**
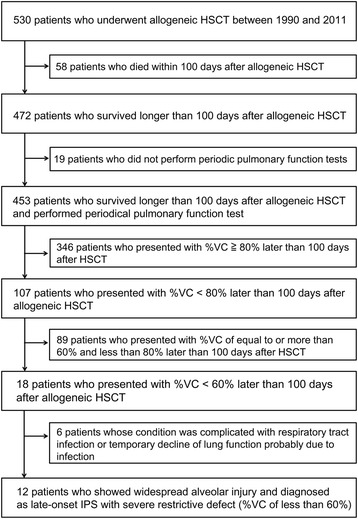
Flowchart of patient enrollment. HSCT denotes hematopoietic stem cell transplantation and VC denotes vital capacity

We reviewed, in the institutional database, the medical records of 530 consecutive patients who underwent allogeneic HSCT at Keio University Hospital (Tokyo, Japan) between January 1990 and December 2011. The patient enrolment flowchart is shown in Fig. 1. Twelve (2.6%) of 453 patients were enrolled in the analysis as patients with late-onset severe restrictive lung defect according to % vital capacity (VC, <60%). There were 89 patients with a restrictive defect at %VC < 80%. Among the 89 patients, there were 86 patients with a restrictive defect at %VC 70–80%; their radiological findings were relatively mild and not specific. In addition, there were 3 patients with a restrictive defect at %VC 60–70%; the three patients showed very mild or no respiratory symptoms. Therefore, only patients with a severe restrictive defect at %VC < 60% were finally enrolled, because we aimed to exclude heterogeneous cases of mild to moderate restrictive ventilatory defect.

### Radiological evaluation

Chest radiographs and chest CT images were evaluated independently by two pulmonologists and one radiologist. The conclusions were reached by consensus.

The radiological features were mainly categorized into 3 patterns: PPFE, unclassifiable interstitial pneumonia (IP), and airway-predominant (centrilobular nodules with BO), based on the consensus of the two pulmonologists and one radiologist.

### Pulmonary function tests

Pulmonary function tests were performed according to established methods [12, 13].

### Diagnosis of acute and chronic graft-versus-host-disease

Acute and chronic graft-versus-host-disease (GVHD) was diagnosed and graded according to established consensus criteria [14, 15].

### Statistical analysis

Differences were analyzed for statistical significance using a paired *t*-test. *P*-values less than 0.05, two-tailed, were considered statistically significant.

## Results

### Clinical features of patients with late-onset severe restrictive lung defect

The clinical features of the patients with late-onset severe restrictive lung defect are summarized in Table 1. The median age at stem cell transplantation was 41 years. Ten patients underwent HSCT from an unrelated donor. Conditioning regimens were total body irradiation (TBI) (12 Gy)-based myeloablative regimens (*n* = 10), busulphan-based myeloablative regimen (*n* = 1), and fludarabine-based regimen in combination with melphalan (*n* = 1).

**Table 1:** Clinical features of patients who underwent hematopoietic stem cell transplantation complicated with severe late-onset idiopathic pneumonia syndrome

Case	Sex	Primary disease	Age at HSCT	Type of donor/Stem cells	HLA compatibility	Conditioning regimen	GVHD prophylaxis	Grades of acute GVHD	Type of chronic GVHD [1]	Organs of chronic GVHD	Months from HSCT to Dx of chronic GVHD	Smoking history	History of Infections	Out-come
1	M	ALL	48	UR/BM	DR serological 1 locus mismatch	TBI (12 Gy) CY	TAC + MTX	II	Extensive	M, E, L	7.0	–	CMV Retinitis Adenovirus cystitis Bacterial pneumonia	Dead
2	F	MDS	43	UR/BM	complete match	TBI (12 Gy) CyA	TAC + MTX	II	Extensive	S, M, E, L	9.4	–	Bacterial pneumonia Influenza virus bronchitis	Alive
3	M	AML	12	UR/BM	complete match	TBI (12 Gy) CyA, CY	TAC + MTX	II	Extensive	S, M, E, L	4.4	–	MRSA pleuritis	Dead
4	F	ALL	53	UR/BM	complete match	TBI (12 Gy) CyA, CY	TAC + MTX	I	Extensive	S, M	3.7	–	CMV enterocolitis	Alive
5	F	CML	35	UR/BM	DRB1 1 allele mismatch	TBI (12 Gy) CyA, CY	TAC + MTX	III	Extensive	S, M, E, G	4.1	–	–	Dead
6	F	ALL	32	R/BM	complete match	BU, CY	CyA + MTX	0	Extensive	E, L	4.8	–	Aspergillus pneumonia Listeria bacteremia	Dead
7	M	MM	39	UR/BM	DRB1 1 allele mismatch	Flu, L-PAM	TAC + MTX	I	Extensive	S, E	2.2	–	Bacterial pneumonia HHV6 encephalomyelitis	Dead
8	F	ALL	49	R/BM	complete match	TBI (12 Gy) CyA, CY	CyA + MTX	III	Extensive	M,G	4.9	–	Pseudomonas pneumonia CMV infection (unknown focus)	Dead
9	M	AML	31	UR/BM	DRB1 1 allele mismatch	TBI (12 Gy) CyA	TAC + MTX	II	Extensive	S, M, E	4.0	–	Bacterial pneumonia, CMV infection (unknown focus)	Alive
10	M	AML	44	UR/BM	A 1 allele mismatch	TBI (12 Gy) CyA	TAC + MTX	I	Extensive	S, M, E, L	4.5	+	Cholecystitis CMV infection (unknown focus)	Dead
11	F	CML	33	UR/ BM	complete match	TBI (12 Gy) CyA	TAC + MTX	I	Extensive	S, M, E, L	3.2	–	Bacterial pneumonia	Alive
12	M	ALL	43	UR/BM	complete match	TBI (12 Gy) CyA, CY	TAC + MTX	III	Extensive	S, M, E, L	46.4	–	–	Dead

*M* male, *F* female, *ALL* acute lymphocytic leukemia, *MDS* myelodysplastic syndrome; *AML* acute myeloid leukemia, *CML* chronic myelogenous leukemia, *MM* multiple myeloma, *HSCT* hematopoietic stem cell transplantation, *UR* unrelated, *R* related, *BM* bone marrow cells, *HLA* human leukocyte antigen, *TBI* total body irradiation, *CY* cyclophosphamide, *CyA* cyclosporine A, *BU* busulphan, *Flu* fludarabine, *L-PAM* melphalan, *GVHD* graft-versus-host disease, *TAC* tacrolimus, *MTX* methotrexate, *M* mouth, *E* eye, *L* liver, *S* skin, *G* gastrointestinal tract, *Dx* diagnosis, *HHV6* human herpesvirus 6

For GVHD prophylaxis, all patients received cyclosporine A or tacrolimus in combination with short-term methotrexate (Table 1). Eleven of 12 patients developed acute GVHD (grade I [*n* = 4], grade II [*n* = 4], and grade III [*n* = 3]). All 12 patients had chronic GVHD and developed the extensive-type (Table 1).

Eight of 12 patients died at a median of 33.5 months after the diagnosis of late onset severe restrictive lung defect (Tables 1 and 2). Seven of 8 patients (87.5%) died of pulmonary or systemic infection (bacterial (*n* = 4); fungal (*n* = 2), and both (*n* = 1)). One died of relapse of multiple myeloma.

**Table 2:** Characteristics of cases with severe late-onset restrictive lung defect developed after hematopoietic stem cell transplantation

Case	Months from HSCT to Dx of LONIPCs	Months of survival from Dx of LONIPCs	Months from HSCT to respiratory symptoms	PPFE pattern	Unclassifiable IP pattern	Airway-predominant pattern (Centrilobular nodules with BO)	Bronchiectasis	BO	Pneumothorax	Pneumomediastinum
1	90	42	103	+	–	–	+	–	–	–
2	26	>84	67	+	–	–	+	–	+	–
3	41	132	62	+	–	–	+	–	+	–
4	40	>33	54	+	–	–	–	–	+	–
5	109	60	109	+	–	–	–	–	–	–
6	60	16	64	–	+	–	–	–	+	–
7	16	3	16	–	+	–	–	+	+	–
8	22	44	19	–	–	+	–	+	–	–
9	50	>80	69	–	–	+	+	+	–	–
10	126	10	124	–	–	+	–	+	–	–
11	36	>117	30	+	–	–	–	+	–	–
12	48	25	54	+	–	–	+	–	+	+

*HSCT* hematopoietic stem cell transplantation, *Dx* diagnosis, *LONIPCs* late-onset noninfectious pulmonary complications, *PPFE* pleuroparenchymal fibroelastosis, *IP* interstitial pneumonia, *BO* bronchiolitis

### Characteristics of patients with late-onset severe restrictive lung defect

The median time from transplantation to diagnosis was 44.5 months (Table 2). HRCT demonstrated pleuroparenchymal thickening with volume loss, associated with evidence of fibrosis, in the upper lobe (PPFE pattern) in 7 (58.3%) patients, bronchiectasis in 5 (41.7%) patients, BO in 5 (41.7%) patients, pneumothorax in 6 (50%) patients (Table 2). Among 5 non-PPFE pattern cases, unclassifiable IP pattern was observed in 2 patients (cases No. 6 and No. 7) and centrilobular nodules with BO, associated with the features of airway-predominant diseases (airway predominant pattern), were observed in 3 patients (cases No. 8, No. 9, and No. 10). All 5 patients complicated with BO showed air trapping on expiratory HRCT.

**Fig. 2:**
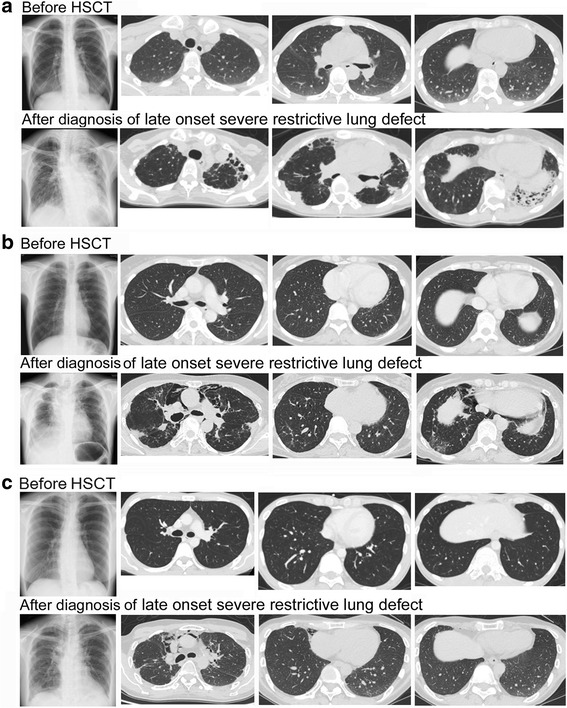
Representative CT findings of PPFE pattern in patients with late-onset severe restrictive lung defect. Chest radiograph and chest CT of representative cases [**a**) case No. 2, **b**) No. 4, and **c**) No. 12] with late-onset severe restrictive lung defect cases before HSCT and after diagnosis of late-onset severe restrictive lung defect are shown

**Fig. 3:**
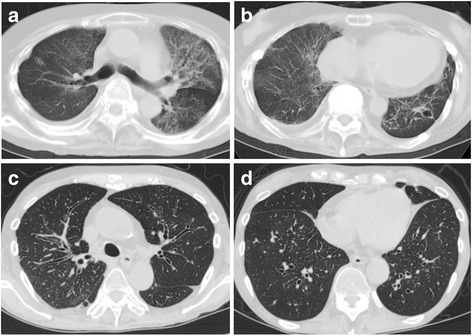
Representative CT findings of non-PPFE pattern in patients with late-onset severe restrictive lung defect. **a**, **b** CT findings of unclassifiable IP in case No. 6 are shown. **c**, **d** CT findings of airway-predominant disease with BO in case No. 8 are shown

Representative chest radiographs and HRCT images of PPFE pattern are shown in Fig. 2a, b, and c. Bronchiectasis was observed in the left lower lobe in case No. 2 (Fig. 2a). Representative CT findings of unclassifiable IP pattern in case No. 6 (Fig. 3a, b) and airway-predominant pattern (i.e., diffuse bilateral bronchial wall thickening and centrilobular nodules) in case No. 8 (Fig. 3c, d) are also shown.

### Pulmonary function changes in patients with late-onset severe restrictive lung defect

**Fig. 4:**
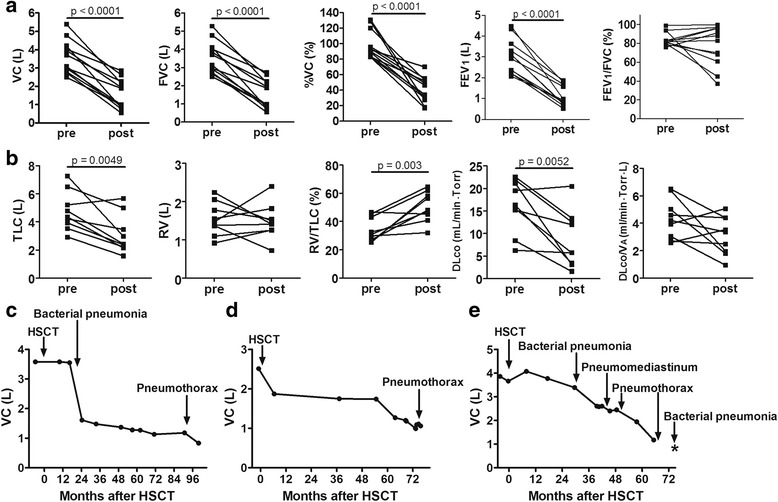
Pulmonary function in patients with late-onset severe restrictive lung defect. **a** Spirometry data including vital capacity (VC), forced vital capacity (FVC), vital capacity as percent of predicted (%VC), forced expiratory volume in one second (FEV_1_), and FEV_1_/ FVC ratio before HSCT (pre) and after (post) diagnosis of late-onset severe restrictive lung defect cases are shown. **b** Data of pulmonary function tests including total lung capacity (TLC), residual volume (RV), RV/TLC ratio, diffusing capacity of the lung for carbon monoxide (DLco), and DLco divided by the alveolar volume (DLco/V_A_) are shown. **c**, **d**, **e** Representative time-course changes of VC in patients with late-onset severe restrictive lung defect. Time course changes of VC in c) case No. 2, d) case No.4, and e) case No. 12 are shown. * indicates death

The results of spirometry are shown in Fig. 4a. Data presented as post-diagnosis are those at diagnosis (cases No. 8 and No. 10) or those most recently examined (other cases), and data presented as pre-diagnosis were those before HSCT. The median follow up was 105.8 months. The median %VC before HSCT was 91% (range, 83–131%). However, the level of median %VC was dramatically decreased (40%, range 18–55%) after diagnosis of late onset severe restrictive lung defect. The levels of VC, forced vital capacity (FVC), and forced expiratory volume in one second (FEV_1_) were also decreased after HSCT.

The data of lung volumes and DLco were available in 9 of 12 patients excluding cases No. 7, No. 8, and No. 12. Data presented as pre-diagnosis were those before HSCT (cases No. 4, No. 6, and No. 9) or those performed earliest after HSCT at a median of 12.6 months after HSCT (range, 5.0–40.2) but before diagnosis of late-onset severe restrictive lung defect (other cases). Data presented as post-diagnosis were those most recently examined (Fig. 4b). The median follow up was 100.3 (range, 30.5–138.0) months. The level of TLC and DLco was markedly decreased after HSCT, while RV/TLC ratio increased after HSCT. The data of lung volumes and DLco in each radiological pattern were shown in the Additional file 1: Figure S1.

Representative data of time course changes of VC in three patients (cases No. 2, No. 4, and No. 12) whose CT images are presented in Fig. 2 are shown (Fig. 4c, d, and e). In case No. 2, the episode of bacterial pneumonia resulted in marked decrease in VC, followed by further decrease in VC after the episode of pneumothorax (Fig. 4c). In case No. 4, pneumothorax as well as unknown etiology seemed to contribute to decrease in VC (Fig. 4d). In case No. 12, the episode of bacterial pneumonia reduced VC level, and pneumomediastinum and pneumothorax further decreased VC (Fig. 4e). These results indicate that the onset of pneumothorax and pulmonary infection contributed to the progressive decrease in restrictive pulmonary function and exacerbation of late onset severe restrictive lung defect, thus contributing to its poor prognosis.

## Discussion

In this retrospective observational study of allogeneic HSCT recipients, we investigated the clinical features of patients with late-onset severe restrictive lung defect (%VC less than 60%) and identified 12 cases with characteristic radiological findings and pulmonary function changes.

We have shown the incidence of cases of late-onset severe restrictive lung defect (%VC less than 60%) was 2.6% in patients who survived more than 100 days after allogeneic HSCT and underwent spirometry (12 of 453 cases). The incidence of cases of late-onset severe restrictive lung defect developing later than 100 days after HSCT, which has previously been reported as IPS, was 3.5% in adult patients [6]. Another report showed the incidence of cases of late-onset severe restrictive lung defect, which has previously been reported as IPS, as 3.1% in pediatric patients with 1.6% severe restrictive ventilatory defect of %VC less than 60% [16]. The incidence of cases of late-onset severe restrictive lung defect observed in the present study is comparable to those of previous studies. A distinctive characteristic of our retrospective cohort study is that we observed, not only more than 500 consecutive HSCT cases, but also pulmonary function test data on more than 95% of these cases were available. Although this was a retrospective study in a single center, we believe that the HSCT incidence rate and other data are highly reliable and representative.

Late-onset severe restrictive lung defect cases in the present study showed the following characteristic radiological findings: pleuroparenchymal thickening with volume loss, predominantly in the upper lobe (PPFE pattern) in 7 of 12 patients. A previous report demonstrated that PPFE, originally reported as idiopathic [10], had features of late-onset lung involvement after allogeneic HSCT [11]. Although we have not confirmed PPFE by pathological examination in the present study, CT findings in 7 of 12 patients were consistent with the findings seen in patients with PPFE. The other 5 non-PPFE cases were evaluated, and we found that 3 cases were airway-predominant pattern, and 2 cases were unclassifiable IP pattern. Therefore, late-onset severe restrictive lung defect cases in the present study could be categorized into 3 groups based on radiological features: PPFE pattern IP (*n* = 7), airway-predominant pattern with BO (*n* = 3), and unclassifiable IP pattern (*n* = 2). In the present study, half of the patients had a history of pneumothorax, and 5 patients showed BO. Only one of the 5 patients with BO developed pneumothorax. Conversely, PPFE could have contributed to the onset of pneumothorax as subpleural fibrosis is attributed to recurrent rupturing of bullae [11]. Indeed, among 6 cases with a history of pneumothorax, 4 patients showed typical PPFE pattern on HRCT. The 2 other cases were both unclassifiable IP, suggesting that pulmonary fibrosis may be a risk factor for pneumothorax in patients with late-onset severe restrictive lung defect.

With regard to clinical features, we believe there are mainly two clinical courses leading to late-onset severe restrictive lung defect: those associated with and those not associated with BO. Late-onset severe restrictive lung defect associated with BO would display mixed ventilatory impairment after exacerbation of obstructive ventilatory impairment due to BO. In contrast, late-onset severe restrictive lung defect not associated with BO directly displays severe restrictive ventilatory impairment induced by subpleural fibrosis and rupture involving pneumothorax.

The present study demonstrated that VC tended to decrease after pneumothorax and pulmonary infection. There were two exceptional cases (cases No. 8 and No. 10) of airway-predominant diseases, of which %VC was mildly increased after the diagnosis of late-onset severe restrictive lung defect. This may be associated with cases that are not progressive PPFE pattern IP but airway-predominant diseases with BO. TLC and DLco were decreased, while RV/TLC ratio was increased in the present study. A previous report demonstrated that idiopathic PPFE showed increased RV/TLC ratio [17]. This might be due to compensated hyperinflation in the lower lobes, which is not observed in typical idiopathic pulmonary fibrosis. Therefore, increased RV/TLC ratio, in the present study, might also be due to compensated hyperinflation in the lower lobes as well as decreased TLC, especially in patients with PPFE pattern.

It has been reported that extensive chronic GVHD was a risk factor for LONIPCs, which possibly included cases of late-onset severe restrictive lung defect [5]. Consistent with these previous studies, all patients of late-onset severe restrictive lung defect, in the present study, had developed chronic GVHD. These results suggest that chronic GVHD could be a risk factor for late-onset severe restrictive lung defect cases.

The outcomes of patients with late-onset severe restrictive lung defect in the present study were unfavorable [9]; only 4 of 12 patients were alive at the time of the analysis with a median month from diagnosis to death of 33.5 months. Except for one case of death due to the relapse of multiple myeloma, all patients succumbed due to systemic or pulmonary infection, which is a novel finding in patients with late-onset severe restrictive lung defect.

There were limitations in the present study; one is that we were not able to obtain pathological specimens. Therefore, we could not pathologically confirm either PPFE or BO. However, the diagnosis of BO does not necessarily require biopsy per the 2014 NIH consensus development project [18]. Consequently, we do not believe pathological confirmation is essential to diagnose patients with late-onset severe restrictive lung defect, considering the patients’ QOL and the difficulty in performing biopsy. The second is that the range of the timing of the pulmonary function tests may be too wide as pulmonary function tests presented as pre-diagnosis were those before HSCT or those performed after HSCT at a median of 12.6 months after HSCT (range 5.0–40.2 months). The third is that VC levels might not be accurate for identifying the development of late restrictive lung defect, because the patient could have a moderate restrictive lung defect due to early BO. A large, prospective study may contribute to clarifying the late-onset severe restrictive lung defect after HSCT.

We should be more aware of the unique entities after HSCT. Although there are still few reports on these entities, more unrecognized cases are expected to happen in the real clinical setting. Considering from the perspective of HSCT, only 2.4% of HSCT patients proceed to late-onset severe restrictive lung defect, while most of the cases do not proceed to this condition. We speculate that more unknown risk factors related to late-onset severe restrictive lung defect exist. In the future, we should seek more definite risk factors of severe restrictive lung defects and interventions to prevent late-onset severe restrictive lung defect in the early stage post-HSCT by accumulating more cases.

## Conclusion

In summary, we have demonstrated the clinical characteristics of late-onset severe restrictive lung defect cases. The incidence of late-onset severe restrictive lung defect cases reported in the current study is comparable to that previously reported. Major CT findings in late-onset severe restrictive lung defect cases were pleuroparenchymal thickening with volume loss predominantly in the upper lobe, which was consistent with PPFE pattern on HRCT. The major cause of death was systemic or pulmonary infection with respiratory failure. The findings of our study may help to elucidate the unique clinical and radiological features of late-onset severe restrictive lung defect after HSCT.

## Additional file

Additional file 1: Figure S1. Pulmonary function in patients with late-onset severe restrictive lung defect in each HRCT pattern. (JPEG 528 kb)

## Abbreviations

ATS: American Thoracic Society
BO: Bronchiolitis obliterans
BOOP: Bronchiolitis obliterans organizing pneumonia
BOS: BO syndrome
COP: Cryptogenic organizing pneumonia
CT: Computed tomography
DAH: Diffuse alveolar hemorrhage
GVHD: Graft-versus-host-disease
HSCT: Hematopoietic stem cell transplantation
IPS: Idiopathic pneumonia syndrome
LONIPCs: Late-onset noninfectious pulmonary complications
MDS: Myelodysplastic syndrome.
PPFE: Pleuroparenchymal fibroelastosis
TBI: Total body irradiation
