# Supplementary figures and images for: Clinical and radiological characteristics of patients with late-onset severe restrictive lung defect after hematopoietic stem cell transplantation
Source: BMC Pulm Med. 2017 Sep 7;17:123. doi: 10.1186/s12890-017-0466-7 (PMC5590140; doi:10.1186/s12890-017-0466-7)

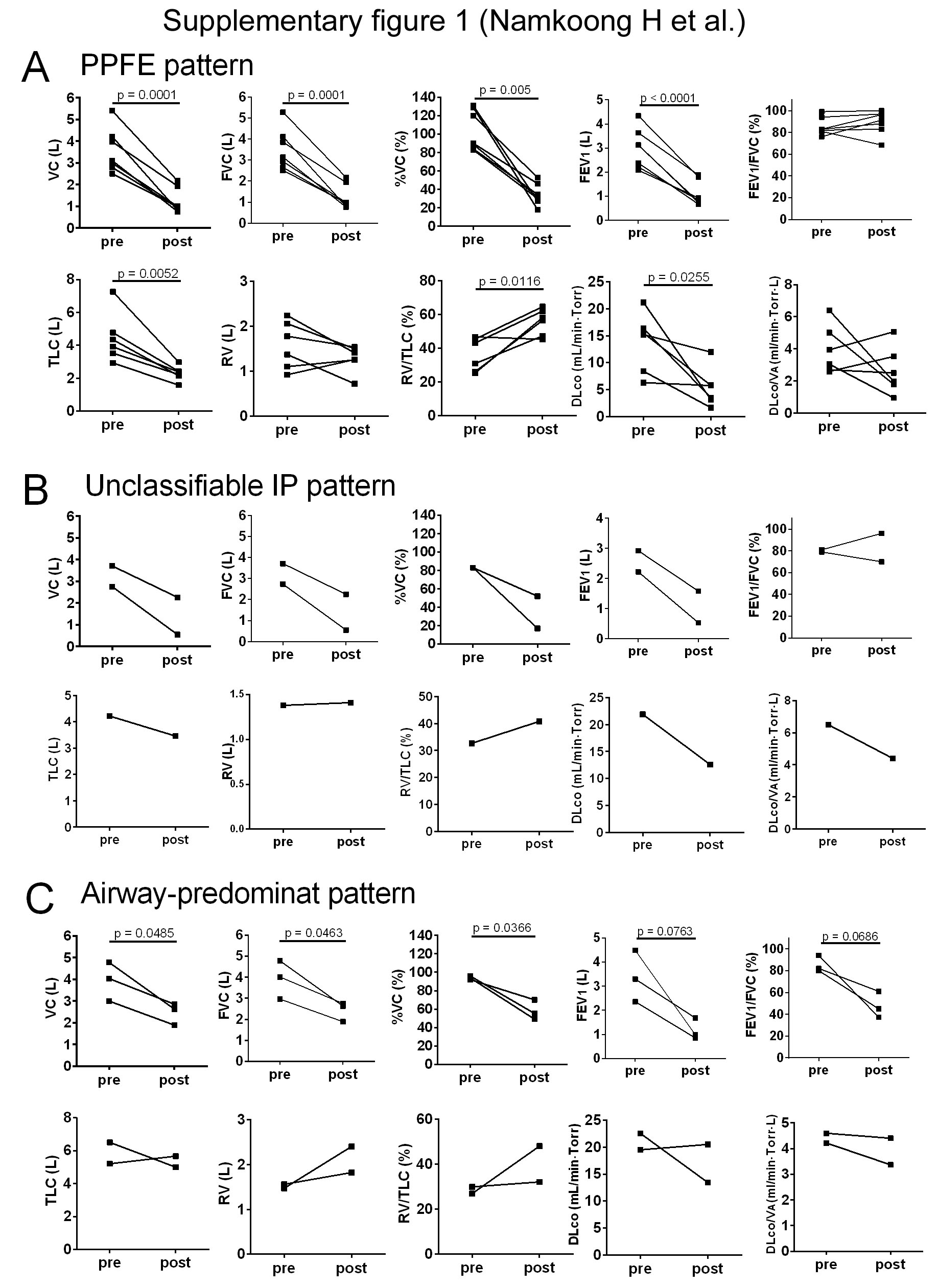

Supplement: Additional file 1: Figure S1. — Pulmonary function in patients with late-onset severe restrictive lung defect in each HRCT pattern. (JPEG 528 kb) [file 12890_2017_466_MOESM1_ESM.jpg]
